# The Interactions between the Antimicrobial Peptide P-113 and Living *Candida albicans* Cells Shed Light on Mechanisms of Antifungal Activity and Resistance

**DOI:** 10.3390/ijms21072654

**Published:** 2020-04-10

**Authors:** Kuang-Ting Cheng, Chih-Lung Wu, Bak-Sau Yip, Ya-Han Chih, Kuang-Li Peng, Su-Ya Hsu, Hui-Yuan Yu, Jya-Wei Cheng

**Affiliations:** 1 Institute of Biotechnology and Department of Medical Science, National Tsing Hua University, Hsinchu 300, Taiwan; s888211@hotmail.com (K.-T.C.); oops801011@gmail.com (C.-L.W.); neuron.hch@gmail.com (B.-S.Y.); a_pu58120@hotmail.com (Y.-H.C.); richard850210@gmail.com (K.-L.P.); miki503285@gmail.com (S.-Y.H.); gwalt1103@gmail.com (H.-Y.Y.); 2 Department of Neurology, National Taiwan University Hospital Hsinchu Branch, Hsinchu 300, Taiwan

**Keywords:** antimicrobial peptide, *Candida albicans*, protease, non-natural amino acid, NMR

## Abstract

In the absence of proper immunity, such as in the case of acquired immune deficiency syndrome (AIDS) patients, *Candida albicans*, the most common human fungal pathogen, may cause mucosal and even life-threatening systemic infections. P-113 (AKRHHGYKRKFH), an antimicrobial peptide (AMP) derived from the human salivary protein histatin 5, shows good safety and efficacy profiles in gingivitis and human immunodeficiency virus (HIV) patients with oral candidiasis. However, little is known about how P-113 interacts with *Candida albicans* or its degradation by *Candida*-secreted proteases that contribute to the fungi’s resistance. Here, we use solution nuclear magnetic resonance (NMR) methods to elucidate the molecular mechanism of interactions between P-113 and living *Candida albicans* cells. Furthermore, we found that proteolytic cleavage of the C-terminus prevents the entry of P-113 into cells and that increasing the hydrophobicity of the peptide can significantly increase its antifungal activity. These results could help in the design of novel antimicrobial peptides that have enhanced stability in vivo and that can have potential therapeutic applications.

## 1. Introduction

*Candida albicans* is the most common human fungal pathogen that can be isolated from the oral and vaginal mucosa and the gastrointestinal tract [1,2]. In healthy individuals, the fungus is commensal and does not cause significant disease. However, in the absence of proper immunity, such as in patients with immunodeficiency, the fungus can cause mucosal and even life-threatening systemic infections [3]. With the significant growth in the population exhibiting oral and systemic candidiasis, there is a great need for the development of novel antifungal agents.

P-113 (AKRHHGYKRKFH), a 12-amino-acid peptide derived from histatin 5, retains antifungal activity comparable to that of the parent molecule [4]. It is active against clinically important microorganisms such as *Pseudomonas* spp., *Staphylococcus* spp., and *C. albicans* [4,5]. Recently, a clinical study on human immunodeficiency virus (HIV) patients showed that P-113 has a positive result for oral candidiasis therapy [6]. Another study on the application of P-113 to gingivitis showed its safety and efficacy in a clinical study [7]. The proposed mechanism of the candidacidal activity of P-113 is similar to that of histatin 5. Initially, the positively charged residues of P-113 bind to the negatively charged *C. albicans* surface through electrostatic interactions, followed by binding to the cell-wall protein Ssa2 and translocation to the cytoplasm [8]. Ssa proteins belong to the heat-shock protein 70 (HSP70) family with roles in heat shock protection, protein folding assistance, and translocation across membranes [9]. In addition, Ssa1p and Ssa2p play important roles in cell-mediated immune responses in mice and humans infected by *C. albicans* [10]. The two cationic amino acids Lys2 and Lys10 of P-113 play important roles in transport into the cytosol [8]. The efficacy of P-113 is greatly reduced at high salt concentrations [11].

Despite the promising results of P-113 as antifungal, *Candida* can become resistant to antimicrobial peptides by producing antimicrobial peptide (AMP)-degrading proteases. Specifically, *Candida* produces secreted aspartic proteinases (Saps), which are also suggested to function as virulence factors [12]. There are 10 Sap proteinases, encoded by a family of 10 *SAP* genes, which account for all of the extracellular proteolytic proteins produced by *C. albicans*. The size of the Sap1–10 proteins is between 35 and 50 kDa, and they contain Lys/Arg (KR) or Lys/Lys (KK) processing sites along with four conserved cysteine residues [13]. Sap1 to Sap8 are secreted to the extracellular environment, while Sap9 and Sap10, which play roles in cell surface integrity and cell separation, are located in the cell membrane/wall via a glycosylphosphatidylinositol (GPI) anchor [14]. It was reported that Saps are able to inactivate a vast number of host defense proteins such as salivary lactoferrin, immunoglobulins [15], LL-37 cathelicidin [16], and kininogen-derived NAT26 and HKH20 [17]. Recently, degradation of histatin 5 with Saps derived from *C. albicans* was shown. Sap9 is mainly responsible for the degradation of histatin 5 at physiological pH [18]. In addition, at optimal pH conditions, histatin 5 can be cleaved by other Saps [19]. The C-terminal end of dibasic (KR, KK) or monobasic (K, R) residues of histatin 5 seemed to be the preferred cleavage sites of Sap9 and Sap10 [13]. Despite the extensive information on the interactions between Saps and histatin 5 in vitro, the in vivo interaction between *C. albicans* and AMPs, such as P-113 with potent antifungal activity, is not fully understood.

To improve the resistance of antimicrobial peptides to hydrolysis, several studies developed antimicrobial peptides with modifications that can reduce their sensitivity to proteases; these include adding N-terminal acetylation and C-terminal amidation, replacing d-amino acids at specific positions, and introducing peptidomimetics to increase half-lives [4,20,21]. Furthermore, increasing the hydrophobicity of peptides by conjugating with an acyl chain at their termini and aromatic amino acid end-tags were effective in conferring them stability against proteolytic degradation. Recently, we found that histidine residues in P-113 substituted with bulky unnatural amino acids, such as Nal (β-naphthylalanine), β-diphenylalanines (Dip), and β-(4,4′-biphenyl)alanines (Bip), boost their salt resistance and serum proteolytic stability [11].

Here, we used solution nuclear magnetic resonance (NMR) methods to elucidate the molecular mechanism of interactions between P-113 and living *C. albicans* cells. We also characterized the functional roles of the amino-acid residues of P-113 in this interaction. Furthermore, we investigated the anti-*Candida* activity and mechanism of these bulky amino acids replaced peptides to identify whether they could be translocated to cytosol or localized into membranes.

## 2. Results

### 2.1. Interactions with C. albicans Causes Chemical Shift Changes in P-113 over the Course of 24 Hours

**Figure 1:**
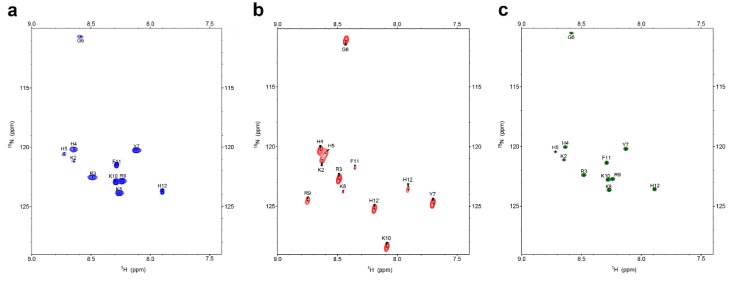
^1^H-^15^N HSQC NMR spectra of 0.25 mM P-113 peptides presented in (**a**) broth only, (**b**) 10^7^ colony-forming units (CFU)/mL of *Candida Albicans*, and (**c**) 10^7^ CFU/mL of *C. albicans* + 0.5 mM pepstatin A at 301 K for 24 h. The chemical shifts of P-113 peptides moved dramatically after *C. albicans* titration. However, these shifts were inhibited by the protease inhibitor pepstatin A.

To explore the molecular mechanism of the interactions between P-113 and living *C. albicans* cells, ^1^H-^15^N HSQC NMR spectroscopy was used to monitor the changes in each amino acid of ^15^N-, ^13^C-labeled P-113 at different time points. The amide chemical shifts of P-113 moved dramatically in the 24 h after the addition of *C. albicans* (Figure 1a,b). To determine whether the cross-peak signals on ^1^H-^15^N HSQC are from P-113 located inside the cell, cells were harvested and resuspended in fresh medium. However, there was no signal from the cell pellet due to low signal-to-noise ratios (data not shown). Recently, Meiller et al. reported that histatin 5 could be inactivated through the hydrolytic action of Saps from *C. albicans* cells [18]. Pepstatin A, an aspartic protease inhibitor, was added with P-113 to inhibit the degradation by *C. albicans*. Interestingly, there were no noticeable changes in ^1^H-^15^N HSQC chemical shifts in the presence of pepstatin A (Figure 1a,c). These results suggest that the chemical shift perturbations of P-113 were caused by Saps-mediated degradation.

### 2.2. Characterization of P-113 Degradation Fragments by NMR

**Figure 2:**
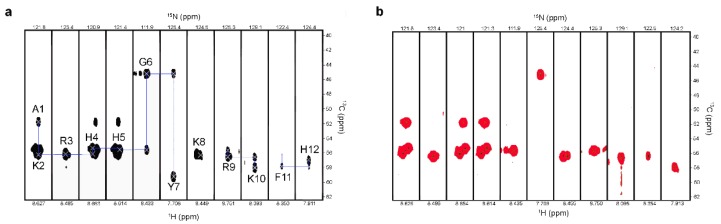
Strip plots representative of three-dimensional, amide-detected heteronuclear NMR spectra for backbone assignment. Inter- and intraresidual correlations of P-113 residues were obtained from correlating (**a**) an HNCA spectrum to (**b**) an HN(CO)CA spectrum. The blue line indicates the sequential walk. A segment of P-113 after degradation by *C. albicans* at 301 K is shown, depicting lost connectivity between the pairs of residues Y7-K8, K8-R9, and K10-F11.

To observe the connectivity of the P-113 backbone after *C. albicans* titration, the six three-dimensional (3D) NMR experiments, HNCA/HN(CO)CA, HNCACB/HN(CO)CACB, and HN(CA)CO/HNCO were performed to establish the backbone walk connections. We found that the connections of Tyr7-Lys8, Lys8-Arg9, and Lys10-Phe11 were removed due to hydrolysis by *C. albicans* (Figure 2). In addition, there were two C^α^, C^β^, and C^γ^ signals of Phe11 and His12 shown in the 3D NMR experiments, but only one of the amide nitrogen signals of Phe11 was seen in ^1^H-^15^N HSQC.

It was reported that Saps produced from *C. albicans* eliminate the antifungal activities of histatin 5 by cleaving lysine residues [22]. To identify whether the N-terminus or C-terminus of lysine amino acids was cleaved by Saps, a two-dimensional (2D) ^15^N-^13^C CON experiment, which records the correlation between the carbonyl carbon and amide nitrogen of the peptide, was performed in the absence or presence of *C. albicans*.

**Figure 3:**
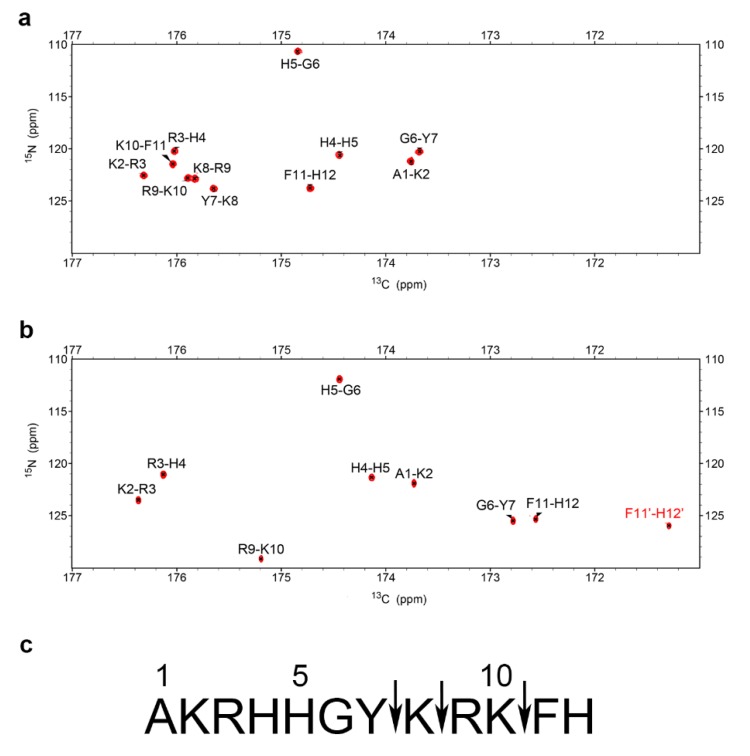
^15^N–^13^C CON spectra of the 0.25 mM P-113 peptides presented in (**a**) Sabouraud dextrose (SD) broth only, and (**b**) 10^7^ CFU/mL of *C. albicans* at 301 K for 24 h. A comparison of the two spectra clearly shows that cross-peaks of Y7-R8, R8-K9, and R10-F11 were lost by degradation, indicating the cleavage sites. (**c**) *C. albicans* proteinase cleavage sites of P-113. Solid arrows indicate the cleavage sites.

P-113 (12 amino acids) contains 11 cross-peaks (peptide bonds, Figure 3a). In contrast, there were only nine cross-peaks in the presence of *C. albicans* likely due to degradation by Saps (Figure 3b). However, the cross-peak between F11 and H12 was divided into two peaks, which meant that there were three cross-peaks (Tyr7-Lys8, Lys8-Arg9, and Lys10-Phe11) lost. The cleavage sites were located at the N-terminus of Lys8 and at the C-terminus of Lys8 and Lys10, giving the four fragments AKRHHGY, K, RK, and FH (Figure 3c).

### 2.3. NMR Time Course Study of P-113 Degradation by C. albicans

**Figure 4:**
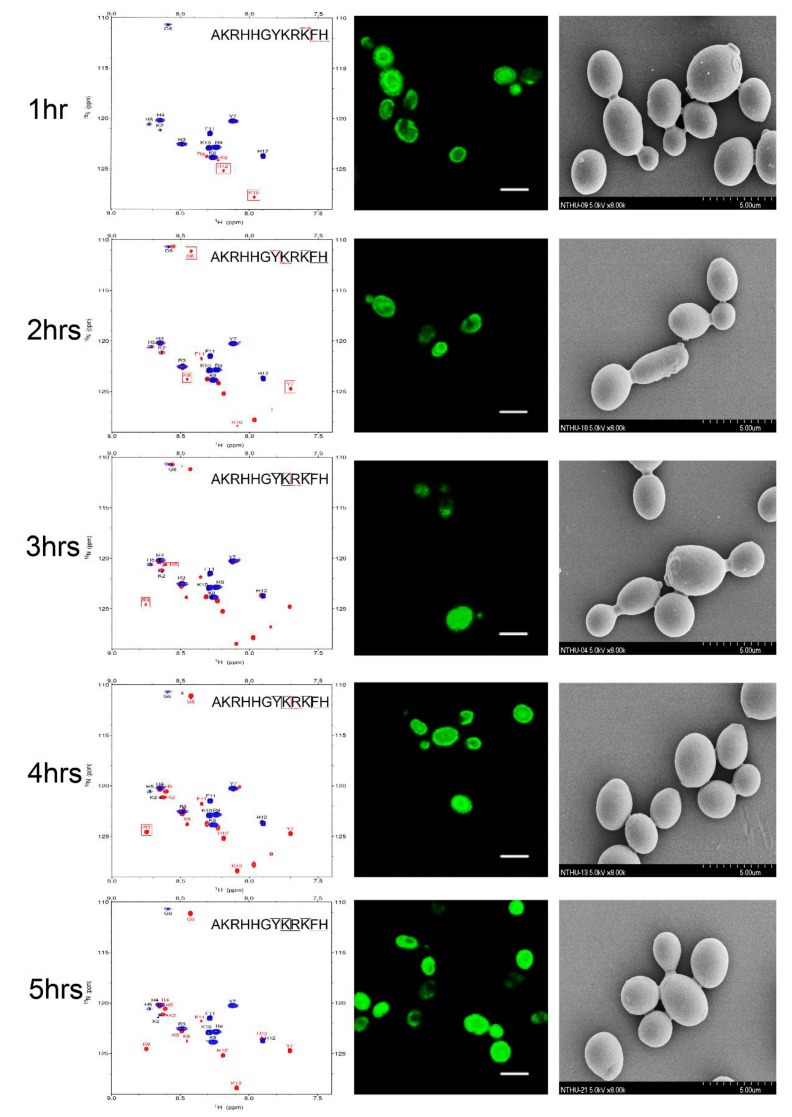
Time-course analysis of 0.25 mM P-113 cleavage following interaction with 10^7^ CFU/mL of *C. albicans* by HSQC spectroscopy (left panels), confocal laser microscopy (middle panels), and scanning electron microscopy (right panels). The bar corresponds to 5 μm. An overlay of ^1^H-^1^5N HSQC spectra for 0.25 mM of P-113 in SD broth only (blue) and broth with *C. albicans* at 301 K at different time points (red) is shown. Perturbations of chemical shifts were observed over 5 h of incubation with *C. albicans* cells. Cell morphology did not change over the 5 h incubation period.

**Figure 5:**
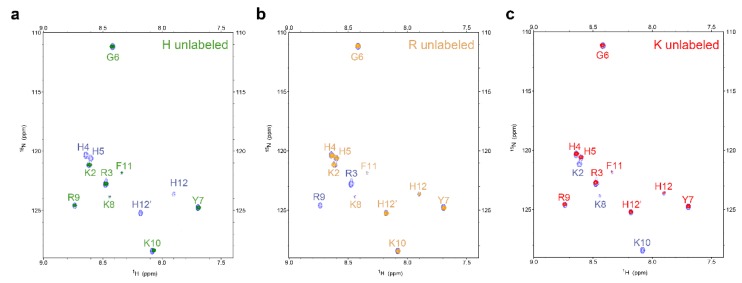
^1^H-^15^N HSQC spectra for the different selectively unlabeled samples of P-113 treated with *C. albicans* at 301 K for 24 h. Blue peaks represent for uniformly ^15^N-labeled P-113: (**a**) green peaks for histidine selectively unlabeled P-113; (**b**) brown peaks for arginine selectively unlabeled P-113; (**c**) red peaks for lysine selectively unlabeled P-113.

**Figure 6:**
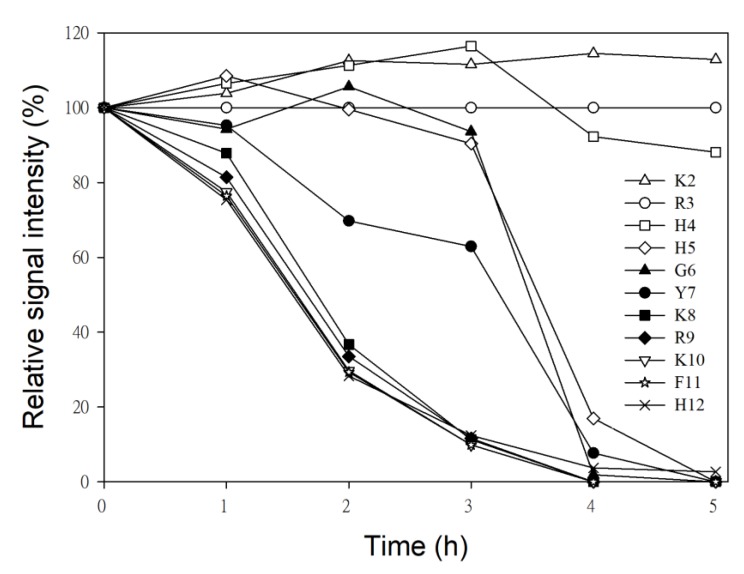
Time-dependent decrease in the intensity of ^15^N-^1^H NMR signals of P-113 residues during incubation with *C. albicans*.

To determine the sequence of cleavage events of P-113, the time-dependent chemical shifts of P-113 incubated with *C. albicans* were recorded by NMR spectroscopy. Confocal and electron scanning micrographs were analyzed at the same time points to determine P-113 translocation and effects on cell morphology (Figure 4). To help identify the chemical shifts of each detected amino acid, the selective ^15^N-unlabeled peptides (Lys, Arg, and His residues) were used (Figure 5). New Lys8, Arg9, Lys10, and His12 cross-peaks were observed after 1 h, indicating that the first cleavage site was between the Lys10 and Phe11 residues. This result was supported by the decrease in intensity of Lys8, Arg9, Lys10, Phe11, and His12 signals (Figure 6).

Therefore, hydrolysis of the Lys10-Phe11 peptide bond occurred first, releasing the C-terminal fragment. Furthermore, new cross-peaks were observed for His5, Gly6, Tyr7, Lys8, and Arg9 after 2–3 h of incubation. These results revealed that the farther cleavage site was between residues Tyr7 and Lys8. The original peaks from His5 to Phe11 with a gradual decay after 4 h of incubation indicated that most of the P-113 peptides were hydrolyzed by this time period. Furthermore, the increasing intensities of the new Arg9 cross-peak indicated that the cleavage site was located between Lys8 and Arg9 residues (Figure 4). Chemical shift perturbations were observed at 5 h of incubation (bottom panel of Figure 4). After 5 h of incubation with *C. albicans*, no further perturbation of chemical shifts was observed, which suggests a near-complete degradation of P-113. Although P-113 degradation started within an hour of incubation, peptides had already translocated into cells, as shown in confocal experiments (Figure 4). The effect of the peptides on cell morphology was also examined by scanning electron microscopy (SEM). The observed cell surfaces were smooth, which indicated that peptides penetrated cell membranes without causing disruption.

### 2.4. Functional Role of N- and C-Terminal Amino Acids in the Antifungal Activity of P-113

Our results suggested that the lysine residues in the P-113 peptide sequence are the targets of cleavage by *C. albicans*. Interestingly, Lys2 is not hydrolyzed by the enzymes, which indicates that the N-terminal lysine has another function. To evaluate whether the N-terminal lysine residue is crucial for the activity of P-113 peptides, we removed the Ala–Lys residues at the N-terminal end of P-113 (N-terminal truncated P-113) and compared it with a P-113 peptide lacking Phe-His residues at the C-terminal end (C-terminal truncated P-113). All peptides were synthesized with fluorescein fluorescent (FITC) tagged at the N-terminal end and amidation at the C-terminal end. The antifungal activities of intact and truncated peptides were examined by two assays: a broth microdilution assay in which the minimum inhibitory concentration (MIC) values were determined and a time killing assay at 1× MIC. The MICs of the truncated P-113s were 8-16-fold higher than that of the full peptide (Table 1).

**Figure 7:**
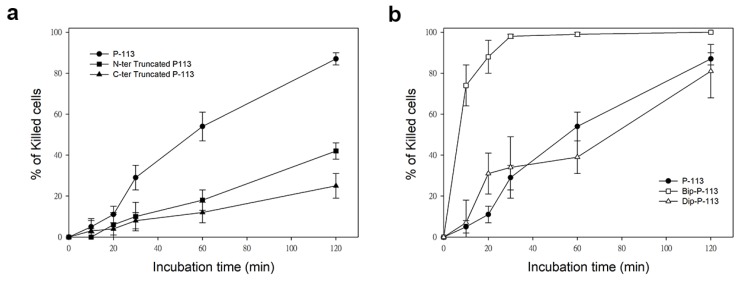
Time killing activity of FITC-labeled P-113 peptide and its FITC-labeled derivatives against *Candida albicans*. Cells were treated with 1× MIC of (**a**) P-113 (black circle), FITC-labeled N-terminal truncated P-113 (black square), FITC-labeled C-terminal truncated P-113 (black triangle), and (**b**) treated with FITC-labeled Bip-P-113 (white square), and FITC-labeled Dip-P-113 (white triangle). Killed cells (%) = (cell number in peptide-free control − cell number in sample)/(cell number in peptide-free control) × 100. Error bars represent the standard errors of the mean.

Similarly, while treatment of *C. albicans* for 120 min with P-113 resulted in 87% cell killing, cell killing by N-terminal and C-terminal truncated peptides was 38% and 25%, respectively (Figure 7). Interestingly, C-terminal truncated P-113, which lacks the first fragment cleaved by *C. albicans*, killed the cells at much slower rates than N-terminal truncated P-113 (Figure 7a). These results, along with the MIC data, offer several insights into the different functional roles of N- and C-terminal amino acids in P-113 peptides.

**Figure 8:**
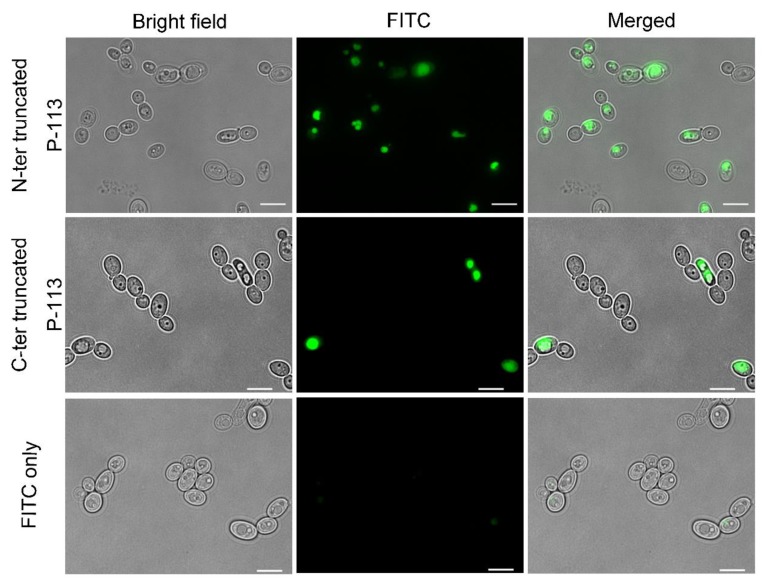
Localizations of FITC truncated P-113 peptides in *C. albicans*. Fluorescence microscopy of *C. albicans* (10^7^ CFU/mL) incubated at 28 °C for 30 min with 50 μM of FITC-truncated P-113 or FITC only. The left panels show a bright field, the middle panels show FITC images, and the right panels show merged images. The bar corresponds to 5 μm.

**Figure 9:**
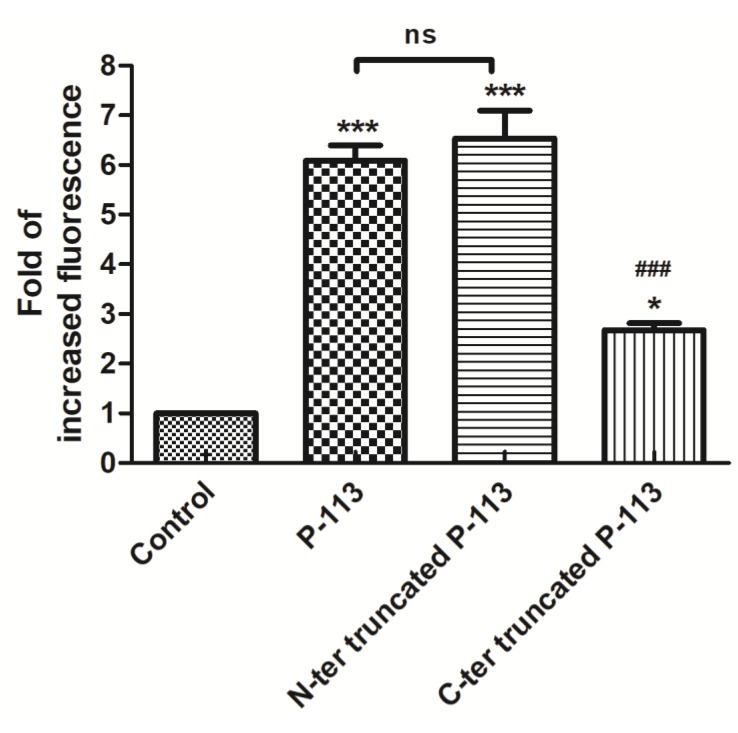
Fluorescence of *C. albicans* FITC P-113, FITC N-terminal truncated P-113, and FITC C-terminal truncated P-113 groups determined by flow cytometry analysis and expressed as fold increase compared to the control group incubated with FITC only. *C. labicans* (5 × 10^6^ CFU/mL) was incubated at 28 °C for 30 min with 50 μM of FITC P-113 or their derivatives. The results are presented as the means ± standard deviations (*n* = 3) of three independent experiments. * *p* < 0.05, *** *p* < 0.001 compared to the control group; ### *p* < 0.001 compared to P-113; ns = no significant difference.

The toxic effect of intact P-113 against *C. albicans* requires transmembrane and intracellular accumulation of the peptide, suggesting that translocation to the cytoplasm might be compromised for the truncated peptides. To determine if this was the case, the cellular localization of N-terminal and C-terminal truncated P-113 peptides was observed by fluorescence microscopy using FITC-labeled peptides. After 30 min of incubation, both truncated P-113 peptides showed a similar localization pattern (Figure 8). However, compared to the N-terminal truncated P-113, less green fluorescence was observed in cells incubated with C-terminal truncated P-113 peptides. Quantification of fluorescence using flow cytometry showed a dramatic decrease in fluorescence intensity in cells incubated with C-terminal truncated peptides compared to that in cells incubated with intact P-113 (Figure 9). These results indicate that the Phe-His residues at the C-terminus of P-113 play an important role in activating peptide translocation, while Ala-Lys residues at the N-terminal end are core sequences for candidacidal activity.

### 2.5. Substituting His 4, 5, and 12 of P-113 for Non-Natural Bulky Amino Acids Altered the Anti-Candida Mechanism

**Figure 10:**
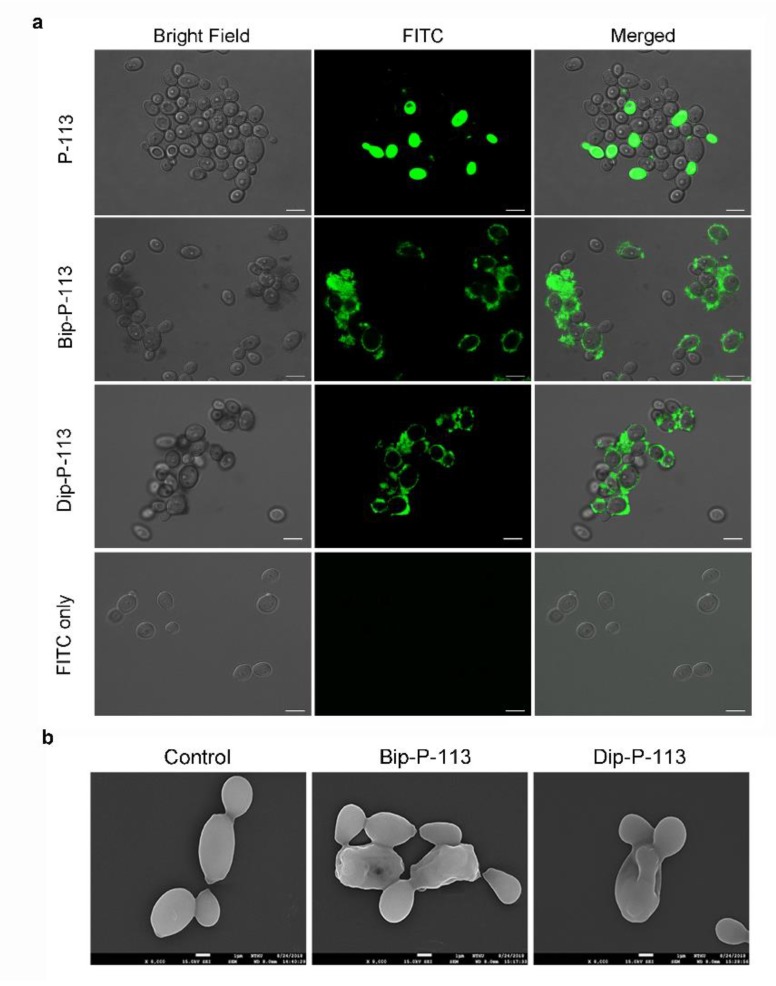
(**a**) Localizations of FITC peptides in in *Candida albicans*. Confocal microscopy was used to visualize 10^7^ CFU/mL of *C. albicans* were incubated at 28 °C for 5 min with 50 μM of FITC-conjugated peptides. The left panels show a bright field, the middle panels show FITC images, and the right panels show merged images. The bar corresponds to 5 μm. (**b**) Scanning electron microscopic micrographs of *C. albicans* only as a control and treated with Bip-P-113 and Dip-P-113 peptides (Bip = β-(4,4β-biphenyl)alanine; Dip = β-diphenylalanine). Each figure is magnified ×8000.

Previously, we found that the replacement of the aromatic residues with bulky aromatic amino acids may generate a potent peptide with improved antimicrobial activity and salt resistance [11]. We synthesized Bip-P-113 and Dip-P-113 by replacing the P-113 histidine residues (His4, His5, and His12) with β-(4,4β-biphenyl)alanine (Bip) and β-diphenylalanine (Dip), respectively. The peptides were conjugated with FITC, and the anti-*Candida* activities of P-113, Bip-P-113, and Dip-P-113 were determined in LYM broth with different salt concentrations (Table 1). Bip-P-113 and Dip-P-113 retained their anti-*Candida* activities under high-salt conditions, while the candidacidal activity of P-113 was reduced by the addition of 100 mM NaCl to the LYM medium. In addition to improved salt resistance, Bip-P-113 exhibited higher killing activity than the other two peptides under normal salt conditions, causing a 99% reduction in cell viability in 30 min, while P-113 and Dip-P-113 exhibited a 87% and 81% reduction after 120 min of incubation, respectively (Figure 7b). Interestingly, Dip-P-113 showed similar kinetics to P-113, which was translocated into the cells and then exhibited killing activity. Membrane disruptions and intracellular translocations of antimicrobial peptides are the two major mechanisms used by candidacidal targets. Next, we studied whether the antimicrobial activity of the bulky amino-acid replaced peptides involved *Candida* cell membrane disruption or translocation into the cytoplasm. Confocal microscopy imaging showed that, while P-113 accumulated in the cytosol after 5 min of incubation with *Candida* cells, Bip-P-113 and Dip-P-113 accumulated on the surface of cells and did not translocate into the cytoplasm (Figure 10a), suggesting that these peptides may exert their effect by direct membrane disruption. Therefore, we evaluated peptide-induced cellular membrane damage by SEM. Untreated *C. albicans* exhibited rough bright and smooth surfaces, whereas the membrane surfaces of *C. albicans* cells exposed to Bip-P-113 or Dip-P-113 for 5 min became rippled and showed deep pore formation (Figure 10b). These results demonstrate that the antifungal activities of Bip-P-113 and Dip-P-113 involve cell membrane disruption.

## 3. Discussion

*Candida albicans* long adapted to human hosts and commonly colonizes the human mucosal surfaces. However, under conditions of immune dysfunction such as those found in HIV+ individuals, *C. albicans* can frequently cause superficial mucosal infections and life-threatening disseminated infections [23]. The virulence of *C. albicans* pathogens appears to correlate with the level of Saps activity that not only facilitates the availability of nutrients for fungal growth, but can also inactivate complement components [24,25,26]. In addition, Saps are reported to neutralize and cleave some human antimicrobial peptides (AMPs) such as LL-37 and histatin 5 [16,18,19]. In-depth investigations of the degradation of P-113, a histidine-rich cationic salivary peptide with strong anticandidal activity, by *C. albicans* are reported in our present study. Here, we demonstrated for the first time the interactions between P-113 and living *C. albicans* cells over time using NMR. In the ^15^N-^1^H HSQC spectra, the chemical shifts of the P-113 peptide dramatically moved after cell titration (Figure 1b). Initially, we expected the chemical shifts to be due to the peptides targeting cell membranes and being imported into cells. Unfortunately, due to low sensitivity and the complexity of living matter, we were not able to demonstrate that the chemical shift perturbations were due to intracellular translocation. Instead, chemical shift movements were caused by hydrolytic cleavage by *C. albicans* proteases (Figure 1). Comparing ^15^N-^13^C CON experiments and backbone assignment of P-113 after degradation, we found that the cleavage sites were located at Tyr7-Lys8, Lys8-Arg9, and Lys10-Phe11, thus producing four fragments (Figure 3c).

Intracellular translocation and accumulation to a threshold concentration in cells are necessary for P-113 to exert candidacidal activity [8]. In a previous study, *C. albicans* cell concentrations of 10^6^ cells/mL and below were shown to be unable to degrade histatin 5 at concentrations of 50–200 μg/mL [18]. However, the hydrolytic level of histatin 5 was proportional to the cell density (>10^7^ cells/mL) and time of exposure. This phenomenon was also observed with P-113 by NMR spectroscopy in the present study. The degradation of P-113 by *C. albicans* resulted in chemical shift perturbations that were recorded in ^1^H-^15^N HSQC spectra. We found that a cell density of 10^7^ cells/mL could degrade 0.25 mM P-113 in five hours. However, chemical shifts did not show any noticeable changes at cell densities below 10^7^ cells/mL (data not shown), indicating lack of insufficient *C. albicans* enzyme levels to fully cleave P-113. That is, complete hydrolysis occurs at the cell-to-peptide ratio of 1:1.5 × 10^13^ (one mole of peptide contains 6.02 × 10^23^ number of peptide molecules). *Candida* concentrations of 600 colony-forming units (CFU)/mL were reported in concentrated rinse samples for healthy commensal carriage [27], with higher levels (above 2–3 × 10^3^ CFU/mL) in individuals predisposed to oral candidiasis [28]. These results suggested that maintaining a concentration of P-113 higher than 0.075 μM (3 × 10^3^ × 1.5 × 10^13^/6 × 10^23^) in the oral cavity is important for controlling the proliferation of commensal *C. albicans* strains. Our findings also explain the clinical trial report that 0.01% P-113 (0.07 μM) in a mouthrinse treatment reduced the development of gingivitis and plaque [29].

The interactions between P-113 and the Ssa proteins were studied by Jang et al. [8]. Lysine residues of P-113 were shown to have specific role in antifungal activity. The substitution of lysine residues at positions 2 and 10 by glutamine results in loss of activity against *C. albicans*, and lack of transport to the cytosol, although it could efficiently bind to the cell wall [8]. Furthermore, the three amino acids Lys-Phe-His of P-113 at the C-terminus of P-113 were suggested to contribute to peptide translocation [30]. However, lysine residues in the histatin 5 sequence are important for its recognition and are prone to be cleaved by the Saps secreted from *C. albicans* cells. In our study, we found that the peptide bond between Lys10 and Phe11 was cleaved by proteases. In contrast, Lys2, at the N-terminus, was able to resist hydrolysis. Thus, we designed N-terminal Ala-Lys-truncated P-113 (RHHGYKRKFH) and C-terminal Phe-His-truncated P-113 (AKRHHGYKRK) peptides to identify the functional role of the N-terminus and the C-terminus of P-113. Although both truncated peptides lost their anti-*Candida* activity, the lower fluorescent intensity level of C-terminal truncated P-113 in the cells suggests that the C-terminal Phe-His residues are an important sequence for active P-113 translocation (Figure 9). This may be the reason why the enzymes from *C. albicans* specifically cut off the C-terminal Phe-His residues of P-113 to prevent peptide entry into the cells via Ssa proteins. This observation can be used to design better antimicrobial peptides to fight against *C. albicans*.

**Figure 11:**
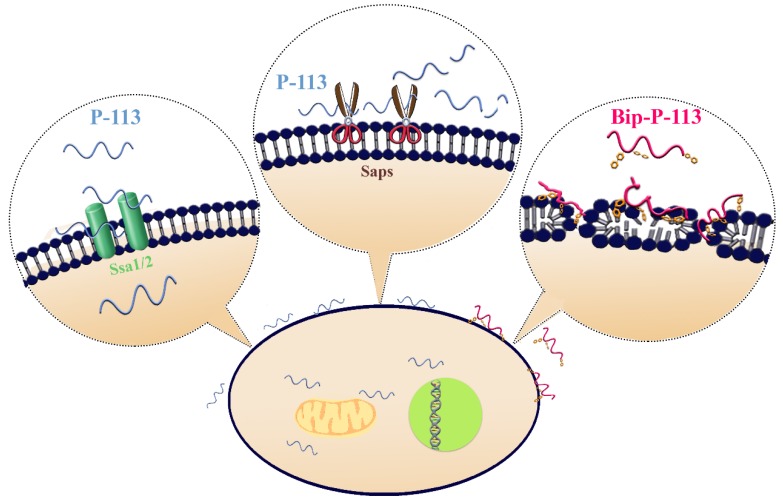
Model of the proposed interactions of P-113 and Bip-P-113 with *Candida albicans*. P-113 peptides recognize and bind to Ssa1/2 proteins and then translocate to the cytoplasm, leading to cell death. However, some of P-113 peptides are degraded by *C. albicans* secreted aspartic proteases (Saps). In contrast, Bip-P-113 accumulates in cell membrane and induces pore formation.

In our previous studies, we found that replacing histidine residues with non-natural amino acids, such as β-naphthylalanine (Nal), β-diphenylalanine (Dip), and β-(4,4′-biphenyl)alanine (Bip), protects Nal-P-113, Dip-P-113, and Bip-P-113 from degradation in serum [31]. To investigate whether Dip-P-113 and Bip-P-113 peptides target to the cell surface instead of translocating into cells, we performed fluorescence microscopic studies to monitor FITC-labeled Dip-P-113 and Bip-P-113 interacting with living *C. albicans* cells (Figure 10a). Our results showed that both Dip-P-113 and Bip-P-113 FITC-labeled peptides localized to the cell surface in 5 min, indicating that Dip-P-113 and Bip-P-113 may possess membrane lytic action. These results are in accordance with the effects observed by SEM, which showed that cells treated with Dip-P-113 or Bip-P-113 had wrinkly surfaces, indicating the loss of membrane integrity leading to pore formation and cell death (Figure 10b). Thus, it seems that introduction of bulky hydrophobic side chains enables the peptide to attack cell membranes. The localization and changes in cell morphology observed with Dip-P-113 or Bip-P-113 suggest that they employ different mechanisms for killing *C. albicans* compared with P-113 (Figure 11). The rapid antimicrobial activity resulting from increased hydrophobicity is suggested to prevent the peptide from being hydrolyzed. Interestingly, Dip-P-113 and Bip-P-113, with the same net charge and hydrophobicity showed differences in killing kinetics (Figure 7b). This could be due to the longer hydrophobic side chain of Bip-P-113 resulting in more efficient insertion and disruption of *C. albicans* membranes compared to Dip-P-113.

In conclusion, we observed the interactions between the salivary anticandidal peptide P-113 and living *C. albicans* cells by NMR spectroscopy. We found that Phe-His residues at the C-terminus of P-113 play a pivotal role in peptide translocation, while the N-terminal Ala-Lys residues are key for anticandidal activity. The resistance strategy of *C. albicans* relies on the proteolytic defensive enzymes that specifically cleaved Phe–His amino-acid sequences, preventing peptides from importing into the cytosol. These findings provide further in-depth information for the design of effective anti-*Candida* agents. Furthermore, elongating the length of the hydrophobic alkyl tails at the position of histidine alters the *C. albicans* killing mechanism of P-113. With its high anti-*Candida* activity, Bip-P-113 deserves further attention in the development of antifungal therapeutics in the future.

## 4. Materials and Methods

### 4.1. Materials

^15^N-labeled ammonium chloride and ^13^C-labeled glucose were purchased from Cambridge Isotope Laboratories, Inc. (Tewksbury, MA, USA). Thermanox substrate-coated cover slips were purchased from Thermo Fisher Scientific (Waltham, MA, USA). Sabouraud dextrose (SD) broth and SD agar were purchased from Becton, Dickinson, and Company (Franklin Lakes, NJ, USA). All other chemicals used in this study were obtained from Sigma-Aldrich (St. Louis, MO, USA).

### 4.2. Peptides

P-113, FITC-conjugated P-113, and P-113 derivatives (>95% purity) were purchased from Kelowna International Scientific Inc. (Taipei, Taiwan).

### 4.3. Yeast Strain

*C. albicans* (ATCC 10231) from the American Type Culture Collection (Manassas, VA, USA) was cultured at 28 °C on SD agar plates.

### 4.4. Nuclear Magnetic Resonance Spectroscopy

To obtain ^15^N-, ^13^C-labeled peptide for three-dimensional NMR analysis, P-113 was expressed in *Escherichia coli* BL21 (DE3) cells grown in M9 minimal media containing ^15^N-labeled ammonium chloride (1 g/L) and ^13^C-labeled glucose (2 g/L). Peptides were purified according to our previous study [32]. All NMR samples were prepared in SD broth containing 10% D_2_O. Sodium 2,2-dimethyl-2-silapentane-5-sulfonate (DSS) was used as an internal chemical shift standard.

For living cell NMR experiments, the interaction between ^15^N-labeled P-113 peptides and *C. albicans* was monitored by 2D heteronuclear single quantum coherence (HSQC) spectroscopy. The peptides were dissolved in SD broth for a total volume of 500 μL at a 0.275 mM concentration. After centrifugation, 10^7^ CFU/mL of cells were resuspended in 50 μL of SD broth and then titrated into the peptide solution. The ^15^N-^1^H HSQC spectra were recorded on a Bruker Avance 600-MHz NMR spectrometer in the States-TPPI mode for quadrature detection at 301 K. The ^1^H and ^15^N carriers were placed at 4.699 and 120 ppm, respectively. After titration, 2D HSQC spectra were collected every hour during 24 h of incubation at 301 K. All spectra were processed with *NMRPipe* and analyzed using the Sparky program (T.D. Goddard and D.G. Kneller, SPARKY3, University of California, San Francisco, CA, USA).

To obtain sequence assignment information, 3D spectra, including those from HNCA, HN(CO)CA, HNCACB, HN(CO)CACB, HN(CA)CO, and HNCO experiments, were recorded on a Bruker Avance 600-MHz NMR spectrometer at 301 K. The ^13^C carriers were placed at 48 ppm for C^α^, at 38 ppm for ^13^C^α/β^ and at 174 ppm for C′. All spectra were processed with *NMRPipe* and analyzed using the Sparky program.

To observe the correlation of amide nitrogen (^15^N_i_) to the carbon (^13^C′) spin in the proceeding residue (^13^C′_i−1_), 2D CON experiments were performed on Bruker Avance 850-MHz NMR spectrometer equipped with a triple-resonance cryogenic probe. The spectra comparing ^15^N-, ^13^C-labeled P-113 in SD broth and in a *C. albicans* titration were recorded with the center frequencies at 120 ppm (^15^N) and 174 ppm (^13^C), respectively. All spectra were processed with *NMRPipe* and analyzed using the Sparky program.

### 4.5. Inhibition of Degradation by Aspartyl Protease Inhibitors

The inhibition of P-113 degradation by *C. albicans* was determined using living cell NMR experiments as described above in the presence of 0.5 mM of the aspartic protease inhibitor pepstatin A.

### 4.6. Expression and Purification of Amino-Acid-Selective ^15^N-Unlabeled P-113

To conveniently observe the change in the chemical shift of each amino acid during the time course experiments, we expressed the selectively unlabeled hG31P-P-113 protein in *E. coli.* The procedures for expressing selectively ^15^N-unlabeled protein were the same as those for the uniformly ^15^N-labeled protein expression. The only difference is the composition of M9 minimal medium, which contains the desired amino acid to be selectively unlabeled at a concentration of 1.0 g/L and together with ^15^NH_4_Cl (0.5 g/L). In the present study, we chose three amino acids for selective unlabeling: Lys (K), Arg (R), and His (H).

### 4.7. Scanning Electron Microscopy Analysis

Time-course morphological studies of *C. albicans* were performed by scanning electron microscopy (SEM). Overnight cultures of *C. albicans* were sub-cultured and grown in SD broth. Then, 10^7^ CFU/mL of *C. albicans* was treated with 0.25 mM P-113 in SD broth at 28 °C. Samples were collected following 1, 2, 3, 4, 5, and 6 h of incubation. Another experiment was performed to observe the morphological changes caused over in a short time caused by Bip-P-113 and Dip-P-113. Briefly, 10^7^ CFU/mL of *C. albicans* in SD broth was treated for 5 min at 28 °C with 50 μM P-113, Bip-P-113, and Dip-P-113. After harvest, cells were resuspended in fixation solution (2.5% glutaraldehyde in 20 mM phosphate buffer pH 7.4) and then placed on Thermanox substrate-coated cover slips at 4 °C for 12 h. The cover slips were carefully washed three times with 20 mM phosphate buffer (pH 7.4), followed by incubation in post-fixation solution (1% OsO_4_ in 20 mM phosphate buffer pH 7.4) at room temperature for an hour. After being washed three times with 20 mM phosphate buffer (pH 7.4), the cover slips were air-dried for an hour by dehydration with a graded series of ethanol. Samples were gold-coated and observed under an S-4700 field-emission (FE)-SEM (Hitachi, Ltd., Tokyo, Japan) at an acceleration voltage of 5 kV.

### 4.8. Confocal Laser Microscopy

Firstly, 10^7^ CFU/mL of *C. albicans* was incubated with 0.25 mM FITC P-113 and 0.25 mM FITC alone (as a negative control) in SD broth at 28 °C for 1, 2, 3, 4, 5, and 6 h. Another experiment was performed to observe the short-term localization of Bip-P-113 and Dip-P-113 peptides. Briefly, log-phase *C. albicans* cultures were resuspended to a cell density of 10^7^ CFU/mL in SD broth and treated for 5 min at 28 °C with 50 μM FITC-conjugated P-113, Bip-P-113, and Dip-P-113. After centrifugation, the cells were washed three times with 20 mM phosphate buffer (pH 7.4) to remove nonbinding peptides and fixed with 4% (*w/v*) paraformaldehyde in 20 mM phosphate buffer (pH 7.4) at room temperature for 20 min. Following fixation, the cells were centrifuged at 3500× *g* for 5 min, resuspended in phosphate buffer, and loaded onto a glass slide with a 1.4% solid agarose bed. The images were taken with a confocal laser scanning microscope (LSM 510 META, Carl Zeiss, Jena, Germany) equipped with a 40× C-Apochromat water-immersion objective lens (Carl Zeiss, Jena, Germany).

### 4.9. Anticandidal Activity Assay

To determine the minimum inhibitory concentration (MIC) values of the FITC conjugated-P-113 peptides and their derivatives, a broth microdilution assay was used. Overnight-grown cultures of *C. albicans* in SD broth were sub-cultured for several hours and subsequently diluted to a final concentration of 5 × 10^4^ CFU/mL in Mueller Hinton (MH) broth and LYM broth. A total of 100 μL of diluted microbe was transferred into each well of a 96-well plate, into which a 1 μL dilution series of peptides in sterile water (ranging from 50 to 0.78 μg/mL) was previously added. After incubation for 24 h at 28 ℃, the MIC value of P-113 peptides was determined as the lowest concentration at which no change in optical density was observed. The experiment was conducted three times, and average values were reported.

### 4.10. Time Killing Assay

*C. albicans* were cultivated onto Sabouraud dextrose agar plates overnight and sub-cultured twice before time killing assays. A total of 5 × 10^4^ CFU/mL of *C. albicans* cells were treated with 1× MIC of FITC-conjugated P-113 peptides and their derivatives in SD broth for 10, 20, 30, 60, and 120 min at 28 °C. After that time, serial dilutions (1:10) of the incubation mixtures were added onto SD agar. The numbers of CFU were counted after 24 h of incubation at 28 °C. The percentage of killed cells was calculated by comparison with the number of cells in a control sample incubated without P-113 peptide. The assays were performed in triplicate.

### 4.11. Fluorescence Microscopy

Firstly, 10^7^ CFU/mL of *C. albicans* cells were incubated with 50 µM FITC N-terminal truncated P-113, FITC C-terminal truncated P-113 peptides, and FITC alone for 30 min at 28 °C in SD broth. After incubation, the cells were centrifuged at 3500× g for 5 min and then washed three times with 20 mM phosphate buffer (pH 7.4) to remove unbound peptides. The resulting pellets were resuspended and immobilized on the glass slips with a 1.4% solid agarose bed. Fluorescence images were captured using an inverted fluorescence microscope (ECLIPSE E400, Nikon, Tokyo, Japan) with a camera (AxioCam ICc5, ZEISS, Jena, Germany).

### 4.12. Flow Cytometry

Firstly, 5 × 10^6^ CFU/mL of *C. albicans* cells were incubated with 50 µM FITC P-113, FITC N-terminal truncated P-113, and FITC C-terminal truncated P-113 peptides for 30 min at 28 °C in SD broth. The resuspended cell pellets described above were also used for flow cytometry experiments. Flow cytometric cell sorting was performed (10,000 events/sample) by using flow cytometry (BD Accuri™ C6 Plus Flow Cytomete, BD Biosciences, NJ, USA). The quantitative data were analyzed by Prism software.

### 4.13. Statistical Analysis

All statistical results are expressed as the means ± standard deviation and were analyzed using one-way ANOVA. Statistical analysis was performed using GraphPad Prism version 5.0, where *p* < 0.05 was considered to indicate a statistically significant difference.

## 5. Conclusions

The chemical shift perturbations of P-113 were caused by Saps-mediated degradation. The cleavage sites were located at the N-terminus of Lys8 and at the C-terminus of Lys8 and Lys10. These results indicate that the Phe-His residues at the C-terminus of P-113 play an important role in activating peptide translocation, while Ala-Lys residues at the N-terminal end are core sequences for candidacidal activity. Furthermore, substituting His 4, 5, and 12 of P-113 for non-natural bulky amino acids increased the killing kinetic and altered the anti-*Candida* mechanism.

## Figures and Tables

**Table 1:** Minimum inhibitory concentration (MIC) values of P-113 and its derivatives against *C. albicans* American Type Culture Collection (ATCC) 10,231 (µg/mL).

Peptide	Sequence	Molecular Weight (Da)	MH Broth	LYM		
				Control	50 mM NaCl	100 mM NaCl
P-113 *	FITC-AKRHHGYKRKFH-NH_2_	2066.36	>50	3.13	25	50
N-ter truncated P-113	FITC-RHHGYKRKFH-NH_2_	1867.14	>50	25	>50	>50
C-ter truncated P-113	FITC-AKRHHGYKRK-NH_2_	1781.49	>50	50	>50	>50
Bip-P-113	FITC-AKR(**Bip**)(**Bip**)GYKRKF(**Bip**)-NH_2_	2324.62	50	6.25	6.25	12.5
Dip-P-113	FITC-AKR(**Dip**)(**Dip**)GYKRKF(**Dip**)-NH_2_	2324.62	>50	12.5	12.5	25

* The MIC of P-113 without the FITC label is the same as the MIC of FITC-P-113.
